# Large-scale proteome profiling identifies circulating biomarkers for disease activity and organ involvement in ANCA-associated vasculitides

**DOI:** 10.3389/fimmu.2026.1848693

**Published:** 2026-06-05

**Authors:** Erik Hellbacher, Ann Knight, Peter Hemmingsson, Anna Juto, Iva Gunnarsson, Annette Bruchfeld, Maria Weiner, Annika Söderbergh, Sophie Ohlsson, Rille Pullerits, Per Eriksson, Christopher Sjöwall, Solbritt Rantapää-Dahlqvist, Johanna Dahlqvist

**Affiliations:** 1 Department of Medical Sciences, Uppsala University, Uppsala, Sweden; 2 Department of Renal Medicine, Karolinska University Hospital and CLINTEC Karolinska Institutet, Stockholm, Sweden; 3 Department of Medicine, Division of Rheumatology, Karolinska Institutet, Stockholm, Sweden; 4 Unit of Rheumatology, Karolinska University Hospital, Stockholm, Sweden; 5 Department of Nephrology, Linköping University, Linköping, Sweden; 6 Department of Health, Medicine and Caring Sciences, Linköping University, Linköping, Sweden; 7 Department of Clinical Nephrology, Region Östergötland, Linköping, Sweden; 8 Department of Rheumatology, Örebro University Hospital, Örebro, Sweden; 9 Department of Nephrology, Division of Clinical Sciences, Lund University and Skåne University Hospital, Lund, Sweden; 10 Department of Rheumatology and Inflammation Research, Institute of Medicine, Sahlgrenska Academy at University of Gothenburg, Gothenburg, Sweden; 11 Department of Clinical Immunology and Transfusion Medicine, Sahlgrenska University Hospital, Gothenburg, Sweden; 12 Department of Biomedical and Clinical Sciences, Division of Inflammation and Infection, Linköping University, Linköping, Sweden; 13 Department of Public Health and Clinical Medicine, Umeå University, Umeå, Sweden

**Keywords:** ANCA-associated vasculitis, biomarker, disease activity, granulomatosis with polyangiitis, microscopic polyangiitis, organ involvement

## Abstract

**Objective:**

Anti-neutrophil cytoplasmic antibody (ANCA)-associated vasculitides (AAV) are chronic, relapsing inflammatory diseases, yet reliable biomarkers for detecting relapse and organ involvement remain limited. This study aimed to identify plasma protein biomarkers that distinguish active disease from remission and to explore markers associated with lung and kidney involvement.

**Methods:**

Plasma samples from 113 patients with granulomatosis with polyangiitis or microscopic polyangiitis (68 active disease and 45 remission) were profiled using a proximity extension assay targeting 181 inflammation- and cardiovascular-related proteins. Clinical data, including CRP and Birmingham vasculitis activity score (BVAS), were collected at sampling. Differential protein expression was assessed using ANOVA, with top candidates validated in independent sample cohorts (plasma samples, *n* = 74; serum samples, *n* = 34). Correlations with BVAS and CRP and discriminatory performance (AUC) were evaluated. Associations with chest- and kidney-specific BVAS were also examined.

**Results:**

A total of 57 proteins were differentially expressed between active disease and remission. Seven proteins (ST2, OPN, IL-2RA, CCL23, IL-6, Flt3L, and SCF) were validated in independent cohorts and showed strong associations with disease activity. Several demonstrated high discriminatory ability (AUC ≥ 0.80) between active disease and remission. Multiple proteins correlated with organ-specific BVAS scores: seven for chest involvement and 16 for kidney involvement, after adjustment for kidney function-related protein variation.

**Conclusion:**

This study identifies a robust panel of plasma proteins that differentiate active AAV from remission and correlate with global and organ-specific disease activity. These biomarkers may enhance non-invasive disease monitoring and support earlier recognition of relapse and organ involvement in AAV.

## Introduction

Granulomatosis with polyangiitis (GPA) and microscopic polyangiitis (MPA) are two anti-neutrophil cytoplasmic autoantibody (ANCA)-associated vasculitides (AAV), characterized by necrotizing inflammation affecting small- and medium-sized blood vessels. ANCAs targeting either proteinase 3 (PR3) or myeloperoxidase (MPO) are found in more than 90% of the patients at some point during the disease course. In AAV, almost any organ may be engaged by inflammation, but kidneys, lungs, and upper airways are most frequently affected. The inflammation often results in tissue damage and chronic organ dysfunction or even complete organ failure.

The disease course of AAV is unpredictable. At disease onset, remission is often effectively induced by cyclophosphamide or rituximab treatment, in combination with corticosteroids. Maintaining remission, however, has proved challenging. Discontinuation of rituximab or the use of traditional disease-modifying antirheumatic drugs in AAV is associated with relapse in up to 30% and 50% of patients, respectively, within 4 years (1–3). Each relapse of vasculitis carries a risk of additional organ damage and dysfunction.

Although certain disease characteristics, such as presence of PR3-ANCA, pulmonary disease involvement, previous relapses, and young age at disease onset, have been associated with an increased risk of relapse (3–5), predicting and detecting relapses remain challenging. With immunosuppressive treatment, AAV patients are prone to infections, with serious infections occurring in up to 50% of the patients within 10 years of diagnosis (6, 7). Yet, distinguishing between relapse and infection in AAV patients remains difficult, as the two scenarios are associated with overlapping symptoms and often with elevated levels of C-reactive protein (CRP) and ANCAs. There is a critical unmet need for reliable biomarkers that can detect relapse in AAV.

Moreover, non-invasive biomarkers for organ-specific disease activity remain limited. At disease onset, identifying inflammatory involvement of organs such as the lungs or kidneys often requires biopsy, as non-invasive findings, such as proteinuria or pulmonary infiltrates, may reflect unrelated comorbid conditions rather than active vasculitis. During follow-up, distinguishing recurrent vasculitis from chronic organ dysfunction caused by prior inflammation or other diseases likewise remains challenging, particularly when proteinuria, microhematuria, or imaging findings fluctuate. Thus, there is a need for improved non-invasive tools capable of identifying organ involvement and enhancing diagnostic precision while reducing reliance on invasive procedures.

With the aim of identifying reliable protein biomarkers for active AAV and for disease involvement of lungs and kidneys, we analyzed the levels of 181 proteins in plasma samples from patients. Top proteins differentiating between patients with active disease and remission were validated in independent cohorts and analyzed for discriminatory ability between active/inactive disease states. Correlations between the 181 proteins and chest and renal components of Birmingham Vasculitis Activity Score (BVAS) (8), respectively, identified candidate biomarkers in patients with active disease.

## Materials and methods

### Cases and controls

For the primary AAV patient cohort, plasma samples were obtained between 2008 and 2023 from patients diagnosed with GPA or MPA across seven rheumatological and/or nephrological centers in Sweden (Umeå University Hospital, Lund University Hospital, Karolinska University Hospital, Linköping University Hospital, Uppsala University Hospital, Örebro University Hospital, Sahlgrenska University Hospital, Gothenburg). In total, samples were collected from 79 patients with GPA and 34 with MPA, fulfilling the classification criteria of the European Medicines Agency algorithm from 2007 for GPA or MPA (9). The patients were sampled either during an active disease state (with a maximum of four days of corticosteroid treatment and no other immunosuppressants) or during remission with ongoing immunosuppressive treatment. As replication cohorts, plasma and serum samples were collected from 74 (GPA *n* = 51, MPA *n* = 23) and 34 (GPA *n* = 23, MPA *n* = 11) distinct AAV patients, respectively, at the mentioned centers. Finally, plasma or serum were obtained from 10 individuals during both active disease and remission. For all cohorts, a majority of patients with active disease were sampled at the time of diagnosis; they had BVAS >0, and the disease activity was assessed as active by the attending physician. Patients in remission were required to have BVAS = 0, a prednisolone dose of maximum 7.5 mg/day, and had not received an induction therapy within the 3 months prior to sampling.

Clinical data were collected from the medical records of all patients and included diagnosis, ANCA subtype, sex, age, and ongoing treatment. BVAS and levels of serum creatinine, CRP, and erythrocyte sedimentation rate (ESR) were recorded at the time of sampling. To calculate the estimated glomerular filtration rate (eGFR), the chronic kidney disease epidemiology collaboration equation (CKD-EPI) (10) was used based on creatinine levels. The characteristics of the patient cohorts are summarized in **Table 1**, **Supplementary Table 1**.

**Table 1:** Baseline characteristics of AAV patients when sampled for proteomic analysis.

Characteristic	Plasma, primary cohort	Plasma, replication cohort	Serum, replication cohort
Sample size, *n*	113	74	34
Age (year), mean (SD)	60 (16)	61 (17)	65 (11)
Sex, female; *n* (%)	51 (45)	30 (41)	13 (38)
CKD-EPI eGFR, mean (SD)	63 (34)	57 (30)	68 (31)
ANCA subtype^a^, *n* (%)			
Proteinase 3	71 (63)	47 (64)	21 (62)
Myeloperoxidase	45 (40)	26 (35)	13 (38)
Diagnosis, *n* (%)			
GPA	79 (70)	51 (69)	23 (67)
MPA	34 (30)	23 (31)	11 (33)
Activity status, *n* (%)			
Active	68 (60)	43 (58)	27 (79)
Remission	45 (40)	31 (42)	7 (21)
Active disease samples			
CRP (mg/L), mean (SD)^b^	41 (46)	26 (44)	105 (79)
ESR (mm), mean (SD)^b^	55 (30)	51 (32)	77 (33)
BVAS, mean (SD)^b,c^	14 (6)^b^	12 (6)	14 (5)
BVAS chest component ≥1, *n* (%)^b,c^	26 (43)	–	–
BVAS renal component ≥1, *n* (%)^b,c^	38/62)	–	–
Disease-modifying drugs at date of sampling, *n* (%)			
No medication	16 (14)	25 (34)	13 (38)
Prednisolone	48 (42)	32 (43)	14 (41)
Methylprednisolone	22 (19)	7 (9)	0
Rituximab	2 (2)	0	6
Azathioprine	8 (7)	8 (11)	0
Methotrexate	7 (6)	7 (9)	2 (6)
Other	7 (6)	5 (7)	0
MD	18 (16)	0	0

ANCA, anti-neutrophil cytoplasmic antibody; AAV, ANCA-associated vasculitis; SD, standard deviation; GPA, granulomatosis with polyangiitis; MPA, microscopic polyangiitis; BVAS, Birmingham Vasculitis Activity Score; CKD-EPI, chronic kidney disease epidemiology collaboration; eGFR, estimated glomerular filtration rate; CRP, C-reactive protein; ESR, erythrocyte sedimentation rate; MD, missing data/patients in remission.

^a^ Three samples were double-positive for PR3- and MPO-ANCA.

^b^ At date of sampling.

^c^ Missing data, *n* = 7.

All data and samples were collected after informed and written consent were obtained from all individuals. The study complies with the Declaration of Helsinki. The regionally appointed ethics committees approved the research protocol.

### Protein measurements

All samples were analyzed for the concentrations of 181 unique proteins using proximity extension assay (PEA) of Olink “Inflammation” and “Cardiovascular III” protein panels (11) at the SciLifeLab Affinity Proteomics Infrastructure Unit in Uppsala, Sweden. The panels include proteins previously associated with immunological processes and cardiovascular disease and were selected due to the inflammatory nature of AAV and the localization to blood vessels. The samples were randomized across 96-well plates, including internal controls, to evaluate technical quality and to normalize protein levels. Assay read-out was provided as Normalized Protein eXpression (NPX), an arbitrary unit on log2-scale where a high value corresponds to a higher protein expression. The samples were analyzed for deviation from the median (>0.3 NPX) of the internal controls as an assessment of quality. Each PEA measurement had a lower detection limit (LOD) calculated based on negative controls; measurements below LOD were removed from further analysis.

### Statistical analyses

The primary cohort and the replication cohorts were analyzed separately, following the same methodology: NPX values were compared between samples from AAV patients with active disease and samples from patients in remission using ANOVA *F*-test, followed by a *post-hoc* test to determine which comparison(s) drove the differential expression (OlinkAnalyze R package (v1.2.4) and the Olink ANOVA posthoc function). Age and eGFR were used as covariates. *P*-values were adjusted for multiple testing using Tukey’s procedure, with adjusted *P <*0.05 set as threshold for significance; the statistical power for the primary cohort was approximately 0.75. Although Olink analysis of plasma may be more informative than that of serum, signature proteins have been shown to largely overlap between plasma and serum samples (12).

To exclude potential analytical interference from ongoing maintenance therapies in patients in remission, all proteins were tested for associations with treatment (methotrexate, azathioprine, rituximab, or mycophenolate mofetil) within this subgroup. Only borderline associations were observed for three proteins (CNTN1, PSP_D, and AXIN1; adjusted *P* = 0.049), while no associations were detected for the remaining proteins (data not shown).

Correlations between the levels of the top seven differentiating proteins (active disease vs. remission) and BVAS, and CRP levels, respectively, were analyzed using Pearson correlation. In patients with active disease in the primary cohort, correlations between all 181 proteins and chest and renal BVAS, respectively, were analyzed using Pearson correlation with adjustment for multiple testing (Benjamini–Hochberg). Analysis of chest involvement was performed with adjustment for eGFR. In addition, Pearson correlation was used to identify proteins correlating with eGFR in patients in remission of the primary cohort.

Receiver operating characteristics (ROC) analysis was performed with all patients of the primary cohort to calculate the area under the curve (AUC) for selected proteins and CRP to discriminate between active disease and remission. To identify the most relevant biomarkers in this selected protein set, step-wise logistic regression was performed to calculate a composite score of the predicted probability. The composite score was used to calculate AUC for a “combined biomarker”, including the most relevant proteins. In addition, ROC analyses were performed with top candidate biomarkers to discriminate between the presence and absence of chest and renal involvement, respectively (chest/renal BVAS ≥ 1 vs. 0), in patients with active disease.

All analyses were performed using R (v.4.1.0–4.5.2) or SPSS (v.30.0.0.0).

## Results

### Identification of candidate biomarkers for active disease

**Figure 1:**
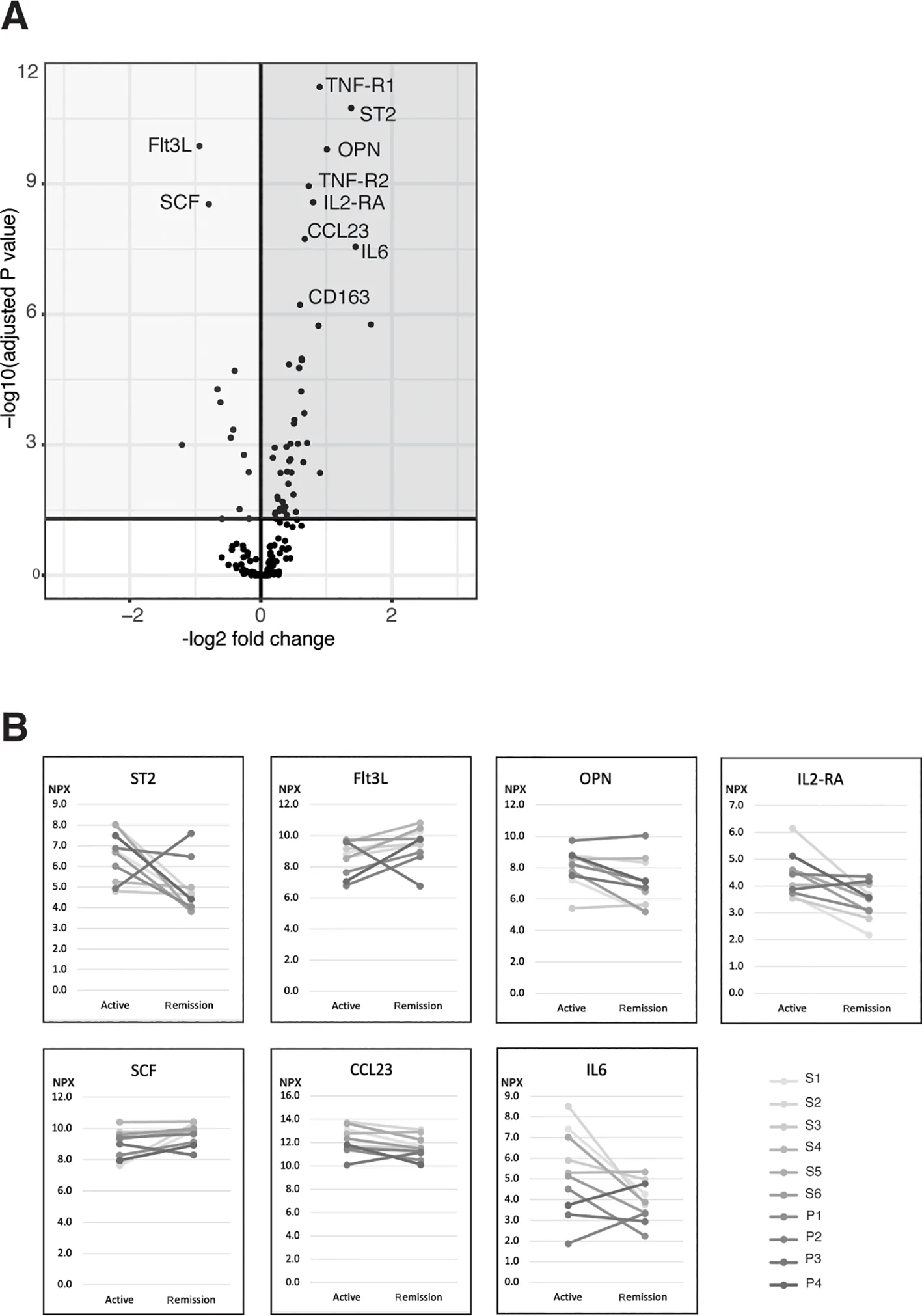
Protein differential expression analysis. **(A)** Volcano plot depicting the differential expression analysis of 181 proteins compared between plasma samples from patients with active AAV and AAV in remission. The top 10 differentiating proteins are specified. **(B)** The levels of seven selected proteins were analyzed in paired samples from 10 individuals sampled at active disease state and remission, respectively. S1–S6, serum samples from six patients; P1–P4, plasma samples from four patients; NPX, normalized protein expression; AAV, anti-neutrophil cytoplasmic antibody-associated vasculitis; ST2, soluble serum stimulation 2; OPN, osteopontin; IL2-RA, interleukin 2 receptor alpha; CCL23, C–C motif chemokine ligand 23; IL6, interleukin 6; Flt3L, FMS-like tyrosine kinase 3 ligand; SCF, stem cell factor; TNF-R1, tumor necrosis factor receptor 1; TNF-R2, tumor necrosis factor receptor 2 (TNF-R2); CD163, cluster of differentiation 163.

In univariate analysis, 57 proteins differed significantly between patients of the primary cohort with AAV in active disease state versus remission (**Supplementary Table 2**). Since there was a substantial overlap of top differentially expressed proteins in patients with PR3-ANCA and MPO-ANCA, respectively (**Supplementary Table 3**), all AAV patients were analyzed jointly. Among the 10 most strongly associated proteins (adjusted *P* < 1 × 10^-7^), eight were elevated in active disease (tumor necrosis factor receptor 1 (TNF-R1), soluble serum stimulation 2 (ST2), osteopontin (OPN), tumor necrosis factor receptor 2 (TNF-R2), interleukin 2 receptor alpha (IL2-RA), C-C motif chemokine ligand 23 (CCL23), interleukin 6 (IL6), and cluster of differentiation 163 (CD163)) and two had higher levels in remission (FMS-like tyrosine kinase 3 ligand (Flt3L), stem cell factor (SCF); **Figure 1A**; **Table 2**; **Supplementary Figure 1**). The differential expression between active disease and remission was confirmed for seven of the top 10 proteins in two independent AAV plasma and serum sample cohorts, respectively (ST2, OPN, IL2-RA, CCL23, IL6, Flt3L, and SCF; **Supplementary Table 4**), and these were, thus, considered as a set of candidate biomarkers. Analyses of paired samples obtained from 10 individuals during active disease and remission overall confirmed the direction of protein level changes for these seven proteins, with some inter-individual variability (**Figure 1B**).

**Table 2:** Top 10 proteins differentiating AAV patients in remission versus active disease.

	Active disease (*n* = 68) vs. remission (*n* = 45)		Correlation to BVAS; AAV active disease	
Protein	Log2 fold change^a^	*P*_adj_	Pearson correlation coefficient	*P*
TNF-R1	0.90	5.9 × 10^-12^	0.50	4.2 × 10^-5^
*ST2*	*1.4*	*1.8 × 10^-11^*	*0.64*	*3.1 × 10^-8^*
*Flt3L*	*-0.94*	*1.4 × 10^-10^*	*-0.34*	*0.0070*
*OPN*	*1.0*	*1.6 × 10^-10^*	*0.53*	*9.2 × 10^-6^*
TNF-R2	0.73	1.1 × 10^-9^	0.47	1.4 × 10^-4^
*IL2-RA*	*0.80*	*2.7 × 10^-9^*	*0.55*	*4.1 × 10^-6^*
*SCF*	*-0.79*	*2.9 × 10^-9^*	*-0.25*	*0.048*
*CCL23*	*0.67*	*1.8 × 10^-8^*	*0.50*	*4.5 × 10^-5^*
*IL6*	*1.4*	*2.8 × 10^-8^*	*0.080*	*0.54*
CD163	0.60	6.0 × 10^-7^	0.39	0.0021

Italicized entries indicate that the data were validated in replication cohorts.

AAV, anti-neutrophil cytoplasmic antibody-associated vasculitis; BVAS, Birmingham Vasculitis Activity Score; *P*_adj_, adjusted *P*-values according to Tukey’s procedure.

^a^ Positive values indicate higher levels in active disease than remission.

### Discriminatory ability and correlations between biomarkers

**Figure 2:**
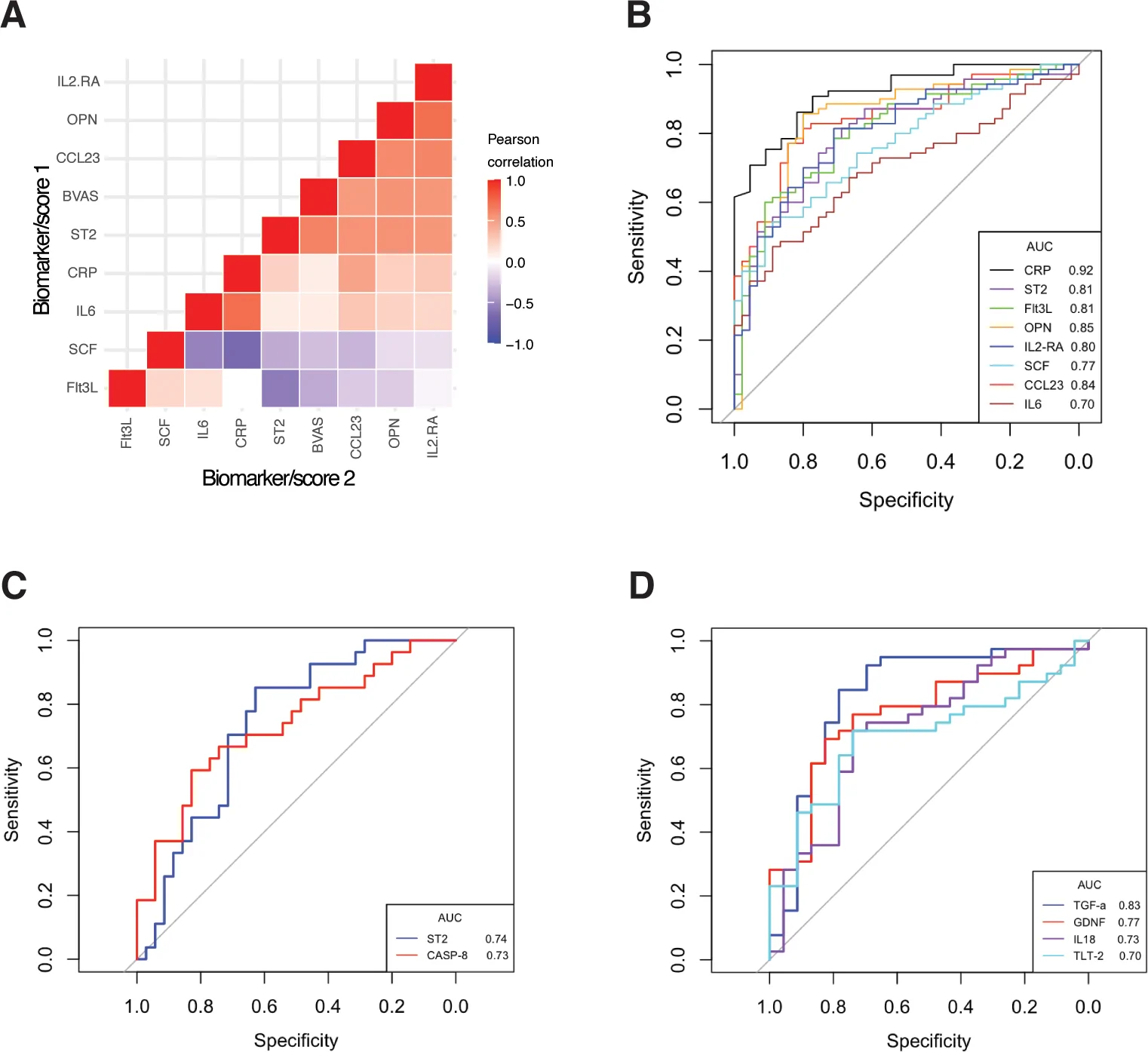
Correlation and ROC analyses. **(A)** Pearson correlation analyses between the top seven candidate biomarkers, CRP, and BVAS. The numbers indicate the Pearson correlation coefficient. **(B–D)** ROC curves and AUC scores illustrating the discriminatory ability of selected proteins, distinguishing between active AAV and remission **(B)**, between the presence and absence of chest involvement in patients with active disease **(C)**, and between the presence and absence of renal involvement in patients with active disease **(D)**. AAV, anti-neutrophil cytoplasmic antibody-associated vasculitis; ROC, receiver operating characteristics; AUC, area under the curve; ST2, soluble serum stimulation 2; OPN, osteopontin; IL2-RA, interleukin 2 receptor alpha; CCL23, C–C motif chemokine ligand 23; IL6, interleukin 6; Flt3L, FMS-like tyrosine kinase 3 ligand; SCF, stem cell factor; CRP, C-reactive protein; BVAS, Birmingham Vasculitis Activity Score.

To explore the relationship between the seven novel candidate biomarkers of active AAV and established assessment tools of disease activity, correlations with CRP levels and total BVAS were calculated. CRP showed the strongest correlations with IL6 and SCF (*r* = 0.69 and *r* = -0.68, respectively), with no or weak correlations with the remaining proteins (*r* = 0.12–0.42; **Figure 2A**). BVAS correlated strongly with the levels of ST2, OPN, IL2-RA, and CCL23 (*r* ≥ 0.50), but weaker with the levels of Flt3L and SCF (*r* = -0.34–0.25) and did not significantly correlate with IL6 (*r* = 0.080; **Figure 2A**, **Table 2**). Additionally, there was no correlation between CRP and BVAS (*r* = 0.059; data not shown). Among the top seven candidate biomarkers, there was a strong correlation between ST2, OPN, IL2-RA, and CCL23 (r > 0.55); in addition, there was a strong inverse correlation between ST2 and FLt3L (*r* = -0.56; **Figure 2A**).

To evaluate the ability of the seven biomarkers to discriminate between active disease state and remission, ROC and AUC were calculated for each individual protein. CRP showed the highest discriminatory ability (AUC 0.92), followed by OPN, CCL23, ST2, and Flt3L (AUC > 0.80) and IL2-RA, IL6, and SCF (AUC 0.70–0.80; *P* < 0.001 for all eight proteins; **Figure 2B**). To investigate whether combining biomarkers would improve AUC in comparison with single protein analysis, the most relevant biomarkers among the seven proteins and CRP were identified using step-wise logistic regression analysis, highlighting CRP, OPN, IL6, and ST2, with a combined AUC of 0.98 (*P* < 0.001; **Supplementary Figure 2**).

### Biomarkers for chest and renal disease involvement

To identify candidate biomarkers for specific organ involvement in active AAV, the levels of the 181 proteins were analyzed for correlations with chest and renal BVAS, respectively, in patients with active disease of the primary cohort (**Table 1**). Seven proteins were identified as significantly correlating with chest involvement (*P*_adj_ ≤ 0.05; IL-12B, CASP-8, DLK-1, IL-1RT2, KLK6, CXCL9, Notch 3, ST2; **Table 3**). ST2 and CASP-8 had AUC >0.70 (*P* < 0.001) in discriminating between the presence and absence of chest involvement (**Figure 2C**). Analysis of active kidney disease in AAV is complex since reduced kidney function affects the levels of a multitude of plasma proteins, with or without ongoing inflammation in renal tissue. Subsequently, we performed a correlation analysis between the 181 proteins and renal BVAS in AAV patients with active disease, without adjustment for eGFR, identifying 76 proteins with significant correlation (**Supplementary Table 5**). Next, a correlation analysis between the eGFR of patients in remission and the levels of the 181 proteins was performed, isolating 93 proteins that correlated significantly with eGFR (**Supplementary Table 6**). Removing the eGFR-associated proteins from the list of proteins correlating with renal BVAS left 16 proteins with a significant correlation with kidney disease activity (*P*_adj_ ≤ 0.05; **Table 3**). Four of these proteins (TGF-α, GDNF, IL18, and TLT-2) had AUC >0.70 (GDNF and TGF-α: *P* < 0.001; IL18: *P* = 0.001; TLT-2: *P* = 0.003) in discriminating between the presence and the absence of kidney involvement (**Figure 2D**).

**Table 3:** Correlation analysis between levels of 181 proteins and AAV chest and kidney involvement, respectively, according to BVAS.

Chest			Kidney		
Protein	Pearson correlation coefficient	*P*_adj_	Protein	Pearson correlation coefficient	*P*_adj_
IL-12B	-0.47	0.023	GDNF	0.52	0.00013
CASP-8	0.47	0.023	TGF-alpha	0.49	0.00032
DLK-1	-0.45	0.030	TLT-2	0.46	0.0010
IL-1RT2	0.43	0.039	PAI	-0.38	0.0080
KLK6	-0.43	0.039	IFN-gamma	-0.37	0.0099
CXCL9	-0.42	0.043	CSTB	0.37	0.012
Notch 3	-0.41	0.045	BLM hydrolase	-0.34	0.022
ST2	0.40	0.052	Flt3L	-0.34	0.022
			Notch 3	0.33	0.026
			IL18	0.33	0.026
			TR	-0.33	0.026
			IL7	-0.32	0.032
			PDGF subunit A	-0.31	0.038
			MCP-4	-0.30	0.046
			IL-17A	-0.30	0.047
			PSP-D	0.30	0.049

BVAS scores for chest and kidney involvement, respectively, for patients with active AAV (*n* = 68) were analyzed for correlations with levels of 181 proteins using Pearson correlation, with adjustment for eGFR for chest involvement. Presented are the proteins with *P*_adj_ <= 0.05; for kidney involvement, proteins significantly associated with eGFR in patients in remission were removed.

AAV, anti-neutrophil cytoplasmic antibody-associated vasculitis; BVAS, Birmingham Vasculitis Activity Score; *P*_adj_, adjusted *P*-values according to Benjamini–Hochberg.

## Discussion

Early detection of relapses and recognition of specific organ involvements remain clinically challenging in the care and follow-up of patients with AAV. In this study, we have identified and validated a set of candidate biomarkers for active disease in AAV and suggest novel minimally invasive biomarkers for lung and kidney involvement.

We used a large-scale screening approach to identify candidate biomarkers for active disease in AAV. We identified a key set of seven biomarkers (ST2, OPN, IL2-RA, CCL23, IL6, Flt3L, and SCF) through the validation of top-performing proteins in independent patient sample cohorts. For most of these candidates, there was a strong correlation with BVAS but not with CRP. A combined analysis of three of the proteins (OPN, IL6, and ST2) and CRP resulted in a very high accuracy of discriminating active disease from remission. Based on these results, we propose this set of seven proteins as candidate biomarkers and a complementary tool to CRP to estimate disease activity in AAV.

Because AAV is characterized by an unpredictable relapsing course, numerous studies have sought to identify clinically useful biomarkers of active inflammation. Most prior works have focused on single candidate proteins, often without comparing their performance to other markers (13–16). Among the candidate biomarkers of the present study, soluble ST2 has previously been suggested as a biomarker for active AAV (17). Additionally, a recent study identified CCL23 as a promising biomarker, achieving high accuracy in distinguishing relapse from remission (18). We have previously shown that CCL23, a cytokine produced by activated neutrophils to promote the recruitment of T cells and monocytes (19), is markedly elevated in active AAV compared with healthy controls, but not in rheumatoid arthritis or systemic lupus erythematosus, supporting CCL23 as a biomarker for AAV (20). In contrast, elevated levels of OPN, IL2-RA, and IL6 have been associated with immune activation in a range of autoimmune disorders, such as rheumatoid arthritis, systemic lupus erythematosus, and Crohn’s disease (21–24). While both elevated and decreased levels of Flt3L have been associated with autoimmune diseases (25, 26), SCF has, in contrast to our results, been found to be elevated in, e.g., Grave’s disease and systemic lupus erythematosus (24, 27).

Only a few studies have evaluated broader biomarker panels in AAV. Monach et al. examined 28 proteins in patients with active disease and remission and later followed a subset longitudinally (28, 29). Of the identified candidate biomarkers, five were analyzed in the present study (MMP-3, IL6, IL8, IL18BP, and OPN), with only IL6 and OPN being validated as associated with active disease. Similarly, Ishizaki et al. identified several candidate serum proteins using mass spectrometry (30). Two of the proteins, CD93 and MMP-9, were included in the current study but did not show a significant association with active disease. We found instead that CD93 correlated with kidney function in remission, suggesting non-inflammatory determinants of its circulating levels. Collectively, these findings underscore the need for replication of candidate biomarkers across independent cohorts to ensure robustness and clinical relevance.

Interestingly, the proteins ST2, OPN, IL-2RA, and CCL23, with strong intercorrelations, also demonstrated a strong association with BVAS, in contrast to CRP, which did not correlate with BVAS. Although ST2, OPN, IL-2RA, and CCL23 each exhibited good discriminatory performance for distinguishing active AAV from remission (AUC: 0.80–0.85), CRP was the single biomarker with the highest individual AUC (0.92). Importantly, the discriminatory performance was further enhanced when biomarkers were combined; the combination of CRP, OPN, IL-6, and ST2 yielded an AUC value of 0.98. Consistent with these findings, Monach et al. reported improved discrimination between active disease and remission when combining multiple biomarkers (CRP, IL-18BP, NGAL, and sIL-2RA; AUC: 0.72) compared with CRP alone (AUC: 0.65) (29). The higher AUC values observed in the present study may reflect the larger proportion of patients with active disease who had not yet initiated corticosteroid treatment as well as the exceptionally high analytical sensitivity of the proximity extension assay used.

Accurately determining organ involvement at AAV onset is essential for assessing disease severity and guiding management. Yet, distinguishing active vasculitis from comorbidity-related findings remains difficult: pulmonary infiltrates may reflect prior infection, and proteinuria may stem from residual kidney damage which may add to tubulointerstitial damage and eGFR loss rather than active kidney inflammation (31). During follow-up, suspected renal flares can likewise be hard to separate from declines in kidney function caused by comorbid disease and associated medications. Consequently, invasive kidney biopsies remain central to diagnosis and may need to be repeated during follow-up.

In this study, we set out to identify plasma biomarkers for lung and kidney involvement in AAV as less invasive diagnostic tools. Based on the large-scale screen of 181 proteins, we suggest eight proteins as potential biomarkers for lung involvement in active AAV (IL-12B, CASP-8, DLK-1, IL-1RT2, KLK6, CXCL9, Notch 3, ST2). Notably, Notch 3 has previously been strongly implicated in lung biology, and both Notch 3 and CXCL9 have been associated with multiple lung diseases, such as interstitial lung disease, chronic obstructive pulmonary disease, and sarcoidosis (32, 33). Interestingly, in acute lung injury, IL-1RT2 proved to be a key neutrophil modulator of inflammation (34), while DLK-1 was found to be crucial for alveolar repair (35).

To identify biomarkers of active AAV glomerulonephritis, it is critical to distinguish changes driven by reduced kidney function from those caused by active renal inflammation. Previous large-scale proteomic work has shown that most circulating proteins correlate with eGFR (36), underscoring the need for rigorous adjustment. In this study, all proteins associated with eGFR during remission were excluded from consideration, narrowing the list to 16 candidate biomarkers of renal vasculitis. Among the top markers, IFN-γ has been established as a key inflammatory mediator in experimental MPO-ANCA glomerulonephritis, with IL-18, another identified biomarker, serving as a potent inducer of IFN-γ (37, 38). IL-17, also highlighted in our analysis, is known to be highly expressed in infiltrating immune cells in kidney tissue in AAV glumorulonephritis (39) and plays an essential role in disease development in AAV animal models, where IL-17 deficiency prevents glomerulonephritis (40).

Taken together, these findings reveal a set of plasma proteins with strong biologic plausibility and prior links to lung and kidney pathology, supporting their potential utility as biomarkers of active organ involvement in AAV. In addition, IL-17 and the other molecules may constitute plausible targets in therapeutic trials.

This study benefits from a highly sensitive protein analysis platform, a broad proteomic panel, adjustment for kidney function, access to organ-specific BVAS data, and replication of findings in independent cohorts. In addition, a majority of patients with active disease were sampled before initiation of immunosuppressive treatment. However, several limitations should be noted. Most samples from patients with active disease were collected at disease onset rather than during relapse, limiting the ability to identify relapse-specific biomarkers. Only a small number of longitudinal paired samples were available, reducing insight into within-patient biomarker dynamics, an area that will benefit from additional long-term follow-up analyses. Furthermore, this study included only patients of Swedish origin, restricting generalizability to patient groups of other origins and emphasizing the need for validation in such cohorts. Finally, because distinguishing relapse from infection is a key clinical challenge, the lack of samples from patients with infections restricts evaluation of biomarker specificity in this context. In future studies, these and additional biomarkers will be explored for the distinction between relapse and infection.

In conclusion, using a systematic proteomic strategy, we have identified plasma protein signatures that align with global disease activity in AAV, and we propose minimally invasive indicators of lung and kidney involvement. These proposed biomarkers complement, rather than replace, conventional clinical measures and offer a path to more nuanced disease monitoring. Detection of early biochemical shifts preceding overt relapse will enable refined timing of treatment escalation or de-escalation.

## Data Availability

The datasets presented in this study can be found in online repositories. The names of the repository/repositories and accession number(s) can be found in the article/**Supplementary Material**.
