# Supplementary material for: Large-scale proteome profiling identifies circulating biomarkers for disease activity and organ involvement in ANCA-associated vasculitides
Source: Front Immunol. 2026 Jun 5;17:1848693. doi: 10.3389/fimmu.2026.1848693 (PMC13279738; doi:10.3389/fimmu.2026.1848693)
Supplement: Supplementary file 1 [file DataSheet1.pdf]

Large-scale proteome profiling identifies circulating biomarkers for disease activity and organ involvement in ANCA-associated vasculitides

Authors

Erik Hellbacher, Ann Knight, Peter Hemmingsson, Anna Juto, Iva Gunnarsson, Annette Bruchfeld, Maria Weiner, Annika Söderbergh, Sophie Ohlsson, Rille Pullerits, Per Eriksson, Christoffer Sjöwall, Solbritt Rantapää-Dahlqvist, Johanna Dahlqvist

Supplementary material

<u>Tables</u>

Supplementary Table 1: Baseline characteristics of ten AAV patients sampled both during active disease and during remission.

Supplementary Table 2: All analyzed proteins comparing patient with active AAV and patients in remission.

Supplementary Table 3: Top ten proteins differentiating between active AAV and remission in PR3-ANCA positive and MPO-ANCA positive patients, respectively.

Supplementary Table 4: Replication of top ten proteins differentiating between active AAV and remission, in independent patient groups.

Supplementary Table 5: Proteins with significant correlation with AAV kidney involvement according to BVAS in patients with active disease.

Supplementary Table 6: Proteins with significant correlation with eGFR in AAV patients in remission.

<u>Figures</u>

Supplementary Figure 1: Plasma levels of top ten proteins differentiating between active disease and AAV in remission.

Supplementary Figure 2: ROC analysis of combined biomarker based on four individual proteins, for discrimination between active AAV and remission.

Supplementary Table 1. Baseline characteristics of AAV patients sampled both during active disease and during remission.

Characteristic	Paired sampling: active disease and remission
Sample size, n	20
Plasma/serum samples, n	8/12
No. of distinct individuals	10
Age (year), mean (SD)	62 (11)
Sex, females n (%)	5 (50)
ANCA subtype, n (%)	
Proteinase 3	6 (60)[a]
Myeloperoxidase	5 (50)[a]
Diagnosis, n (%)	
GPA	6 (60)
MPA	4 (40)
Sample activity status, n (%)	
Active	10 (50)
Remission	10 (50)
BVAS, mean (SD)	
Active disease samples	17 (6)
Remission samples	0
Disease-modifying drug at date of sampling, n (%)	
No medications	6 (30)
Prednisolone	11 (55)
Methylprednisolone	3 (15)
Rituximab	0
Azathioprine	4 (20)
Methotrexate	3 (15)
Mycophenolate mofetil	3 (15)
Other	0

[a]One individual double-positive for PR3- and MPO-ANCA.

ANCA = anti-neutrophil cytoplasmic antibody; AAV = ANCA-associated vasculitis; scRNA sequencing = single-cell RNA sequencing; PBMC = Peripheral blood mononuclear cells; SD = standard deviation; GPA = granulomatosis with polyangiitis; MPA = microscopic polyangiitis; BVAS = Birmingham Vasculitis Activity Score.

Supplementary Table 2. All analyzed proteins comparing patient with active AAV and patients in remission.

Protein	OlinkID	UniProt	Panel	Log2 fold change	P_{adj}
TNF-R1	OID00649	P19438	Olink CARDIOVASCULAR III	0.90	5.90E-12
ST2	OID00634	Q01638	Olink CARDIOVASCULAR III	1.38	1.81E-11
Flt3L	OID00533	P49771	Olink INFLAMMATION	-0.94	1.35E-10
OPN	OID00621	P10451	Olink CARDIOVASCULAR III	1.01	1.61E-10
TNF-R2	OID00567	P20333	Olink CARDIOVASCULAR III	0.73	1.13E-09
IL2-RA	OID00570	P01589	Olink CARDIOVASCULAR III	0.80	2.66E-09
SCF	OID00500	P21583	Olink INFLAMMATION	-0.79	2.93E-09
CCL23	OID00530	P55773	Olink INFLAMMATION	0.67	1.85E-08
IL6	OID00482	P05231	Olink INFLAMMATION	1.44	2.81E-08
CD163	OID00577	Q86VB7	Olink CARDIOVASCULAR III	0.60	6.05E-07
NT-proBNP	OID00131	NA	Olink CARDIOVASCULAR III	1.68	1.71E-06
IGFBP-2	OID00650	P18065	Olink CARDIOVASCULAR III	0.88	1.83E-06
PGLYRP1	OID00623	O75594	Olink CARDIOVASCULAR III	0.62	1.05E-05
TNF	OID05548	P01375	Olink INFLAMMATION	0.63	1.13E-05
PD-L1	OID00518	Q9NZQ7	Olink INFLAMMATION	0.43	1.42E-05
U-PAR	OID00620	Q03405	Olink CARDIOVASCULAR III	0.59	1.71E-05
TWEAK	OID00555	O43508	Olink INFLAMMATION	-0.40	1.99E-05
PON3	OID00642	Q15166	Olink CARDIOVASCULAR III	-0.66	5.26E-05
RETN	OID00603	Q9HD89	Olink CARDIOVASCULAR III	0.62	5.89E-05
TRANCE	OID00521	O14788	Olink INFLAMMATION	-0.61	0.00011
MCP-3	OID00474	P80098	Olink INFLAMMATION	0.66	0.00019
TIMP4	OID00585	Q99727	Olink CARDIOVASCULAR III	0.51	0.00026
CCL16	OID00654	O15467	Olink CARDIOVASCULAR III	0.50	0.00032
CNTN1	OID00586	Q12860	Olink CARDIOVASCULAR III	-0.42	0.00045
CCL11	OID00505	P51671	Olink INFLAMMATION	-0.46	0.00069
CHI3L1	OID00633	P36222	Olink CARDIOVASCULAR III	0.71	0.00091
VEGFA	OID00472	P15692	Olink INFLAMMATION	0.45	0.00095
TNFRSF9	OID00553	Q07011	Olink INFLAMMATION	0.57	0.00095
IFN-gamma	OID05547	P01579	Olink INFLAMMATION	-1.20	0.0010
IL-18R1	OID00517	Q13478	Olink INFLAMMATION	0.39	0.0011
CSF-1	OID00562	P09603	Olink INFLAMMATION	0.21	0.0012
DNER	OID01213	Q8NFT8	Olink INFLAMMATION	-0.26	0.0017
SPON1	OID00599	Q9HCB6	Olink CARDIOVASCULAR III	0.18	0.0020
CD40	OID00542	P25942	Olink INFLAMMATION	0.46	0.0021
HGF	OID00522	P14210	Olink INFLAMMATION	0.44	0.0024
GDF-15	OID00595	Q99988	Olink CARDIOVASCULAR III	0.65	0.0025
LTBR	OID00583	P36941	Olink CARDIOVASCULAR III	0.40	0.0041
EGFR	OID00637	P00533	Olink CARDIOVASCULAR III	-0.18	0.0042
MMP-10	OID00527	P09238	Olink INFLAMMATION	0.47	0.0043
CD5	OID00531	P06127	Olink INFLAMMATION	0.30	0.0044
EN-RAGE	OID00541	P80511	Olink INFLAMMATION	0.90	0.0044
TNFRSF14	OID00563	Q92956	Olink CARDIOVASCULAR III	0.42	0.0079
MEPE	OID00132	Q9NQ76	Olink CARDIOVASCULAR III	0.50	0.014
IL-6RA	OID00602	P08887	Olink CARDIOVASCULAR III	0.25	0.016
IGFBP-7	OID00638	Q16270	Olink CARDIOVASCULAR III	0.26	0.018
LIF	OID00547	P15018	Olink INFLAMMATION	0.33	0.020
TGF-alpha	OID00503	P01135	Olink INFLAMMATION	0.35	0.025
CDCP1	OID00476	Q9H5V8	Olink INFLAMMATION	0.37	0.027
IL-18BP	OID00640	O95998	Olink CARDIOVASCULAR III	0.29	0.030
CD6	OID00499	P30203	Olink INFLAMMATION	-0.32	0.030
IL-24	OID00524	Q13007	Olink INFLAMMATION	0.35	0.033
EPHB4	OID00569	P54760	Olink CARDIOVASCULAR III	0.28	0.033
FGF-23	OID00507	Q9GZV9	Olink INFLAMMATION	0.54	0.035
PLC	OID00582	P98160	Olink CARDIOVASCULAR III	0.22	0.036
IL-15RA	OID00514	Q13261	Olink INFLAMMATION	0.22	0.039
CCL3	OID00532	P10147	Olink INFLAMMATION	0.40	0.041
CDH5	OID00587	P33151	Olink CARDIOVASCULAR III	-0.18	0.050
SLAMF1	OID00502	Q13291	Olink INFLAMMATION	0.23	0.050
Ep-CAM	OID00610	P16422	Olink CARDIOVASCULAR III	-0.59	0.050
FABP4	OID00589	P15090	Olink CARDIOVASCULAR III	0.55	0.053
TNFRSF10C	OID00594	O14798	Olink CARDIOVASCULAR III	0.29	0.061
IL8	OID00471	P10145	Olink INFLAMMATION	0.40	0.068
MMP-1	OID00510	P03956	Olink INFLAMMATION	0.62	0.073
IL10	OID00528	P22301	Olink INFLAMMATION	0.48	0.077
CCL15	OID00629	Q16663	Olink CARDIOVASCULAR III	0.27	0.14
TFF3	OID00573	Q07654	Olink CARDIOVASCULAR III	0.37	0.16
MCP-1	OID00484	P13500	Olink INFLAMMATION	-0.37	0.19
IL-17RA	OID00566	Q96F46	Olink CARDIOVASCULAR III	0.21	0.20
LDL receptor	OID00564	P01130	Olink CARDIOVASCULAR III	-0.27	0.21
CXCL16	OID00601	Q9H2A7	Olink CARDIOVASCULAR III	0.16	0.21
CXCL6	OID00534	P80162	Olink INFLAMMATION	-0.43	0.21
Beta-NGF	OID00519	P01138	Olink INFLAMMATION	0.14	0.22
CCL20	OID00556	P78556	Olink INFLAMMATION	0.42	0.24
CSTB	OID00575	P04080	Olink CARDIOVASCULAR III	0.33	0.24
TNFB	OID00561	P01374	Olink INFLAMMATION	-0.26	0.25
PRTN3	OID00618	P24158	Olink CARDIOVASCULAR III	0.39	0.25
FGF-19	OID00545	O95750	Olink INFLAMMATION	-0.44	0.26
TRAIL	OID00488	P50591	Olink INFLAMMATION	-0.20	0.30
CD93	OID00639	Q9NPY3	Olink CARDIOVASCULAR III	0.15	0.30
MB	OID00616	P02144	Olink CARDIOVASCULAR III	0.29	0.31
ICAM-2	OID00646	P13598	Olink CARDIOVASCULAR III	0.15	0.34
CX3CL1	OID00552	P78423	Olink INFLAMMATION	-0.21	0.36
IL-1RT2	OID00627	P27930	Olink CARDIOVASCULAR III	0.18	0.38
TR	OID00593	P02786	Olink CARDIOVASCULAR III	-0.26	0.38
CXCL5	OID00520	P42830	Olink INFLAMMATION	-0.60	0.39
4E-BP1	OID00536	Q13541	Olink INFLAMMATION	0.45	0.41
OSM	OID00494	P13725	Olink INFLAMMATION	0.39	0.41
IL2	OID00495	P60568	Olink INFLAMMATION	-0.07	0.43
CD244	OID00477	Q9BZW8	Olink INFLAMMATION	-0.16	0.47
LIF-R	OID00511	P42702	Olink INFLAMMATION	0.13	0.48
PI3	OID00609	P19957	Olink CARDIOVASCULAR III	0.24	0.48

TR-AP	OID00606	P13686	Olink CARDIOVASCULAR III	0.19	0.50
AXL	OID00612	P30530	Olink CARDIOVASCULAR III	0.12	0.52
MCP-4	OID00504	Q99616	Olink INFLAMMATION	-0.30	0.56
FGF-21	OID00512	Q9NSA1	Olink INFLAMMATION	-0.49	0.57
SHPS-1	OID00628	P78324	Olink CARDIOVASCULAR III	0.14	0.59
CXCL10	OID00535	P02778	Olink INFLAMMATION	-0.38	0.59
CST5	OID00491	P28325	Olink INFLAMMATION	0.19	0.59
IGFBP-1	OID00604	P08833	Olink CARDIOVASCULAR III	-0.37	0.69
GRN	OID00579	P28799	Olink CARDIOVASCULAR III	0.10	0.73
CASP-8	OID00550	Q14790	Olink INFLAMMATION	-0.27	0.73
TFPI	OID00590	P10646	Olink CARDIOVASCULAR III	0.12	0.74
IL-10RB	OID00515	Q08334	Olink INFLAMMATION	0.10	0.75
SELE	OID00596	P16581	Olink CARDIOVASCULAR III	0.16	0.77
MCP-2	OID00549	P80075	Olink INFLAMMATION	-0.23	0.78
ADA	OID00560	P00813	Olink INFLAMMATION	0.15	0.78
FAS	OID00615	P25445	Olink CARDIOVASCULAR III	0.13	0.79
PAI	OID00591	P05121	Olink CARDIOVASCULAR III	-0.28	0.80
JAM-A	OID00625	Q9Y624	Olink CARDIOVASCULAR III	0.28	0.82
BLM hydrolase	OID00581	Q13867	Olink CARDIOVASCULAR III	-0.13	0.83
IL-20RA	OID00489	Q9UHF4	Olink INFLAMMATION	0.10	0.84
IL-12B	OID00523	P29460	Olink INFLAMMATION	-0.24	0.84
IL-10RA	OID00508	Q13651	Olink INFLAMMATION	0.14	0.84
t-PA	OID00635	P00750	Olink CARDIOVASCULAR III	-0.19	0.85
IL-20	OID00537	Q9NYY1	Olink INFLAMMATION	0.10	0.86
CHIT1	OID00605	Q13231	Olink CARDIOVASCULAR III	0.28	0.86
CXCL11	OID00486	O14625	Olink INFLAMMATION	0.23	0.86
Notch 3	OID00584	Q9UM47	Olink CARDIOVASCULAR III	-0.10	0.86
Gal-3	OID00578	P17931	Olink CARDIOVASCULAR III	-0.10	0.86
IL4	OID00546	P05112	Olink INFLAMMATION	0.16	0.87
CCL28	OID00539	Q9NRJ3	Olink INFLAMMATION	-0.09	0.88
SCGB3A2	OID00636	Q96PL1	Olink CARDIOVASCULAR III	0.21	0.89
NT-3	OID00554	P20783	Olink INFLAMMATION	-0.12	0.89
SELP	OID00574	P16109	Olink CARDIOVASCULAR III	0.19	0.89
CXCL1	OID00496	P09341	Olink INFLAMMATION	-0.24	0.90
GP6	OID05026	Q9HCN6	Olink CARDIOVASCULAR III	0.18	0.90
TLT-2	OID00588	Q5T2D2	Olink CARDIOVASCULAR III	0.11	0.90
CTSD	OID00622	P07339	Olink CARDIOVASCULAR III	0.10	0.91
KLK6	OID00647	Q92876	Olink CARDIOVASCULAR III	-0.11	0.92
ST1A1	OID00557	P50225	Olink INFLAMMATION	-0.26	0.92
GDNF	OID00475	P39905	Olink INFLAMMATION	0.09	0.93
MMP-2	OID00614	P08253	Olink CARDIOVASCULAR III	-0.09	0.93
TNFSF13B	OID00617	Q9Y275	Olink CARDIOVASCULAR III	-0.12	0.94
ALCAM	OID00572	Q13740	Olink CARDIOVASCULAR III	-0.05	0.94
uPA	OID00481	P00749	Olink INFLAMMATION	-0.07	0.94
IL-17A	OID00485	Q16552	Olink INFLAMMATION	0.13	0.95
MMP-9	OID00568	P14780	Olink CARDIOVASCULAR III	0.20	0.95
PCSK9	OID00619	Q8NBP7	Olink CARDIOVASCULAR III	0.08	0.96
CCL25	OID00551	O15444	Olink INFLAMMATION	-0.08	0.97
SIRT2	OID00538	Q8IXJ6	Olink INFLAMMATION	0.27	0.97
AXIN1	OID00487	O15169	Olink INFLAMMATION	0.27	0.97
IL-1RT1	OID00613	P14778	Olink CARDIOVASCULAR III	0.05	0.97
ITGB2	OID00565	P05107	Olink CARDIOVASCULAR III	-0.07	0.98
IL-22 RA1	OID00516	Q8N6P7	Olink INFLAMMATION	0.07	0.98
CPA1	OID00624	P15085	Olink CARDIOVASCULAR III	-0.14	0.98
TSLP	OID00497	Q969D9	Olink INFLAMMATION	-0.06	0.98
IL-1 alpha	OID00493	P01583	Olink INFLAMMATION	-0.05	0.98
OPG	OID00479	O00300	Olink INFLAMMATION	0.06	0.99
CXCL9	OID00490	Q07325	Olink INFLAMMATION	0.13	0.99
IL5	OID00559	P05113	Olink INFLAMMATION	0.10	0.99
CCL19	OID00513	Q99731	Olink INFLAMMATION	-0.14	0.99
DLK-1	OID00598	P80370	Olink CARDIOVASCULAR III	-0.08	0.99
TNFSF14	OID00506	O43557	Olink INFLAMMATION	0.10	0.99
IL-2RB	OID00492	P14784	Olink INFLAMMATION	-0.07	0.99
Gal-4	OID00626	P56470	Olink CARDIOVASCULAR III	0.06	0.99
PECAM-1	OID00652	P16284	Olink CARDIOVASCULAR III	0.08	1.00
IL7	OID00478	P13232	Olink INFLAMMATION	0.09	1.00
IL18	OID00501	Q14116	Olink INFLAMMATION	-0.05	1.00
PDGF subunit A	OID00648	P04085	Olink CARDIOVASCULAR III	-0.09	1.00
PSP-D	OID00608	P35247	Olink CARDIOVASCULAR III	0.06	1.00
CPB1	OID00632	P15086	Olink CARDIOVASCULAR III	-0.06	1.00
vWF	OID00651	P04275	Olink CARDIOVASCULAR III	0.08	1.00
LAP TGF-beta-1	OID00480	P01137	Olink INFLAMMATION	-0.05	1.00
AZU1	OID00597	P20160	Olink CARDIOVASCULAR III	0.07	1.00
CD8A	OID05124	P01732	Olink INFLAMMATION	-0.05	1.00
COL1A1	OID00641	P02452	Olink CARDIOVASCULAR III	0.03	1.00
ARTN	OID00526	Q5T4W7	Olink INFLAMMATION	-0.03	1.00
STAMBP	OID00558	O95630	Olink INFLAMMATION	0.08	1.00
NRTN	OID00548	Q99748	Olink INFLAMMATION	-0.02	1.00
CASP-3	OID00630	P42574	Olink CARDIOVASCULAR III	0.09	1.00
IL33	OID00543	O95760	Olink INFLAMMATION	0.01	1.00
CCL4	OID00498	P13236	Olink INFLAMMATION	-0.03	1.00
MPO	OID00600	P05164	Olink CARDIOVASCULAR III	0.03	1.00
MMP-3	OID00644	P08254	Olink CARDIOVASCULAR III	0.05	1.00
RARRES2	OID00645	Q99969	Olink CARDIOVASCULAR III	0.02	1.00
AP-N	OID00611	P15144	Olink CARDIOVASCULAR III	0.01	1.00
IL13	OID00525	P35225	Olink INFLAMMATION	0.02	1.00
CCL24	OID00592	O00175	Olink CARDIOVASCULAR III	0.03	1.00
FGF-5	OID00509	P12034	Olink INFLAMMATION	-0.01	1.00
CTSZ	OID00643	Q9UBR2	Olink CARDIOVASCULAR III	0.01	1.00
IL-17C	OID00483	Q9P0M4	Olink INFLAMMATION	-0.01	1.00

AAV = Anti-neutrophil cytoplasmic antibody-associated vasculitis; P_{adj}= adjusted P values according to Tukey's procedure.

Supplementary Table 3. Top ten proteins differentiating between patients in active disease versus remission in PR3-ANCA positive and MPO-ANCA positive patients, respectively.

PR3-AAV (n=71)			MPO-AAV (n=42)		
**Protein**	**Log2 fold change***	$\mathbf{P_{adj}}$	**Protein**	**Log2 fold change***	$\mathbf{P_{adj}}$
OPN	1.2	1.0×10^{-12}	CCL23	1.0	1.7×10^{-8}
SCF	-1.1	4.5 x 10-^{10}	OPN	1.3	6.2×10^{-8}
ST2	1.3	2.5×10^{-7}	NT-proBNP	2.2	2.6×10^{-6}
IL2-RA	0.7	9.2×10^{-7}	IL2-RA	0.9	4.2×10^{-6}
CCL23	0.7	1.6×10^{-6}	U-PAR	0.8	3.7×10^{-5}
TR-AP	0.9	2.5×10^{-6}	TNF-R1	0.8	4.4×10^{-5}
IGFBP-2	0.8	1.4×10^{-5}	Flt3L	-1.0	1.2×10^{-4}
Flt3L	-0.8	3.7×10^{-5}	ST2	1.3	1.2×10^{-4}
TNF-R1	0.6	6.9×10^{-5}	TNF	1.0	1.4×10^{-4}
IL6	1.6	7.8×10^{-5}	TNFRSF9	0.8	1.8×10^{-4}

***Positive** values indicate higher levels in active disease than remission.
AAV = Anti-neutrophil cytoplasmic antibody-associated vasculitis; PR3 = proteinase 3; MPO = myeloperoxidase; Padj = adjusted P values according to Tukey's procedure.

Supplementary table 4. Replication of the top 10 proteins differentiating between active AAV and remission, in independent patient groups.

	Plasma samples (n=74)		Serum samples (n=34)	
**Protein**	**Log2 fold change***	**P**	**Log2 fold change***	**P**
**TNF-R1**	0.0060	n.s.	0.36	n.s.
**ST2**	0.86	< 0.050	1.1	< 0.050
**Flt3L**	-1.01	< 0.050	-1.3	< 0.050
**OPN**	0.34	< 0.050	1.2	< 0.050
**TNF-R2**	-0.021	n.s.	0.31	n.s.
**IL2-RA**	0.28	< 0.050	1.0	< 0.050
**SCF**	-0.35	< 0.050	-1.2	< 0.050
**CCL23**	0.58	< 0.050	0.72	< 0.050
**IL6**	0.62	< 0.050	1.1	n.s.
**CD163**	-0.022	n.s.	0.26	n.s.

*Positive values indicate higher levels in active disease than remission.
Plasma and serum samples collections were independent.
AAV = Anti-neutrophil cytoplasmic antibody-associated vasculitis.

Supplementary Table 5. Proteins with significant correlation with AAV kidney involvement according to BVAS in patients with active disease.

Protein	Pearson correlation coefficient	P_{adj}
EPHB4	0.736	3.77E-09
GDF-15	0.725	5.38E-09
IGFBP-2	0.710	1.34E-08
CX3CL1	0.696	3.02E-08
TNF-R1	0.692	3.41E-08
IGFBP-7	0.689	3.59E-08
PLC	0.683	4.95E-08
CCL15	0.677	6.74E-08
TNF-R2	0.672	8.23E-08
LTBR	0.669	9.59E-08
IL-10RB	0.655	2.25E-07
IL-15RA	0.653	2.25E-07
TNFRSF14	0.653	2.25E-07
MEPE	0.647	3.16E-07
TFF3	0.645	3.19E-07
CD93	0.641	3.88E-07
MB	0.638	4.56E-07
CD5	0.627	8.75E-07
CTSZ	0.620	1.15E-06
FAS	0.621	1.15E-06
NT-proBNP	0.619	1.18E-06
DLK-1	0.609	2.05E-06
TNFRSF9	0.606	2.36E-06
LIF-R	0.600	3.21E-06
IL-18BP	0.595	4.05E-06
FGF-5	0.586	6.16E-06
SHPS-1	0.582	7.19E-06
SPON1	0.582	7.19E-06
KLK6	0.575	1.00E-05
CST5	0.565	1.60E-05
TIMP4	0.559	2.04E-05
OPN	0.554	2.48E-05
IL-1RT1	0.543	4.15E-05
U-PAR	0.524	9.23E-05
GDNF	0.516	1.29E-04
RETN	0.507	1.81E-04
FABP4	0.500	2.42E-04
CHI3L1	0.492	3.23E-04
TGF-alpha	0.491	3.23E-04
MMP-3	0.484	4.17E-04
Gal-4	0.471	6.50E-04
TLT-2	0.458	1.01E-03
FGF-23	0.456	1.08E-03
PD-L1	0.455	1.09E-03
CD40	0.448	1.37E-03
CCL23	0.446	1.44E-03
AXL	0.435	2.04E-03
CXCL16	0.430	2.28E-03
ST2	0.430	2.28E-03
IL2-RA	0.429	2.29E-03
VEGFA	0.420	3.07E-03
IL-6RA	0.405	4.80E-03
TIMP-1	0.397	5.97E-03
IL-17C	0.395	6.06E-03
PI3	0.395	6.06E-03
CHIT1	0.386	7.72E-03
PAI	-0.384	8.05E-03
CSF-1	0.381	8.62E-03
IFN-gamma	-0.375	9.93E-03
CSTB	0.367	1.22E-02
TNFB	-0.357	1.55E-02

PGLYRP1	0.356	1.56E-02
ALCAM	0.350	1.78E-02
TNFRSF10C	0.347	1.90E-02
BLM hydrolase	-0.340	2.20E-02
Flt3L	-0.340	2.20E-02
Notch 3	0.333	2.60E-02
IL18	0.331	2.64E-02
TR	-0.331	2.64E-02
IL7	-0.322	3.17E-02
IGFBP-1	0.316	3.59E-02
PDGF subunit A	-0.313	3.80E-02
CCL16	0.310	4.05E-02
MCP-4	-0.303	4.62E-02
IL-17A	-0.302	4.68E-02
PSP-D	0.300	4.86E-02

AAV = Anti-neutrophil cytoplasmic antibody-associated vasculitis;
Padj = adjusted P values according to Tukey's procedure.

Supplementary Table 6. Proteins with significant correlation (P =< 0.05) with eGFR in AAV patients in remission.

**Protein**	**Pearson correlation coefficient**	**P**
FABP4	-0.831	<0.001
TNF_R1	-0.801	<0.001
CCL15	-0.785	<0.001
PLC	-0.778	<0.001
CX3CL1	-0.777	<0.001
EPHB4	-0.725	<0.001
LTBR	-0.715	<0.001
IL_15RA	-0.711	<0.001
IL_10RB	-0.709	<0.001
FGF_23	-0.708	<0.001
TNF_R2	-0.704	<0.001
GDF_15	-0.7	<0.001
CXCL16	-0.671	<0.001
DLK_1	-0.665	<0.001
CST5	-0.663	<0.001
IGFBP_7	-0.657	<0.001
FAS	-0.64	<0.001
CTSZ	-0.635	<0.001
TNFRSF9	-0.626	<0.001
TIMP4	-0.623	<0.001
CCL25	-0.622	<0.001
IL_18BP	-0.616	<0.001
IGFBP_2	-0.609	<0.001
CHI3L1	-0.603	<0.001
SPON1	-0.589	<0.001
PI3	-0.582	<0.001
NT_proBNP	-0.577	<0.001
FGF_21	-0.562	<0.001
Notch.3	-0.56	<0.001
MB	-0.558	<0.001
CD93	-0.556	<0.001
OPN	-0.553	<0.001
SCGB3A2	-0.542	<0.001
OPG	-0.54	<0.001
TFF3	-0.535	<0.001
SCF	-0.527	<0.001
SHPS_1	-0.527	<0.001
Gal_4	-0.519	<0.001
LIF_R	-0.517	<0.001
TNFRSF14	-0.505	<0.001
CPB1	-0.498	<0.001
RARRES2	-0.491	<0.001
VEGFA	-0.481	<0.001
IL_1RT1	-0.48	<0.001
IL_18BP	0.476	<0.001
CD93	0.479	<0.001
IGFBP_7	0.504	<0.001
ICAM_2	0.631	<0.001
CPA1	-0.47	0.001
IL2_RA	-0.469	0.001
CCL16	-0.458	0.002
CD40	-0.456	0.002
U_PAR	-0.454	0.002
KLK6	-0.449	0.002
CSF_1	-0.448	0.002
CXCL9	-0.446	0.002
IL_22.RA1	-0.441	0.002
AXL	-0.439	0.003
IGFBP_1	-0.429	0.003
TFPI	-0.429	0.003
CDCP1	-0.427	0.003

SLAMF1	0.427	0.003
KLK6	0.428	0.003
CCL4	0.431	0.003
CCL3	-0.418	0.004
TRAIL	0.422	0.004
IL_17C	-0.415	0.005
RETN	-0.413	0.005
MMP_3	-0.408	0.005
ALCAM	-0.398	0.007
GRN	-0.396	0.007
Beta_NGF	-0.385	0.009
MMP_10	-0.383	0.009
VEGFA	0.384	0.009
PD_L1	-0.375	0.011
PON3	0.372	0.012
TNFRSF10C	-0.368	0.013
MCP_1	0.366	0.013
ST2	-0.363	0.014
PDGF.subunit.A	0.363	0.014
IL_6RA	-0.361	0.015
MEPE	0.357	0.016
CCL23	-0.354	0.017
CHIT1	-0.35	0.019
CD5	-0.348	0.019
IL6	-0.328	0.028
FGF_5	-0.327	0.028
CTSZ	0.322	0.031
RARRES2	0.32	0.032
CD6	0.319	0.033
PGLYRP1	-0.309	0.039
uPA	0.303	0.043
TNFB	0.301	0.044

AAV = Anti-neutrophil cytoplasmic antibody-associated vasculitis;
eGFR = estimated glomerular filtration rate
according to CKD-EPI.
